# Foraging Range of Honey Bees, *Apis mellifera*, in Alfalfa Seed Production Fields

**DOI:** 10.1673/031.011.14401

**Published:** 2011-11-02

**Authors:** James R. Hagler, Shannon Mueller, Larry R. Teuber, Scott A. Machtley, Allen Van Deynze

**Affiliations:** ^1^ Arid Land Agricultural Research Center, USDA-ARS, 21881 North Cardon Lane, Maricopa, AZ, 85138 USA; ^2^ University of California Cooperative Extension, 1720 South Maple Avenue, Fresno, CA 93702 USA; ^3^ University of California, Department of Plant Sciences, Mail Stop I, One Shields Avenue, Davis, CA, 95616 USA

**Keywords:** dispersal, ELISA, fluorescent dust marking, insect mark-capture, pollen-mediated gene flow, protein marking

## Abstract

A study was conducted in 2006 and 2007 designed to examine the foraging range of honey bees, *Apis mellifera* (Hymenoptera: Apidae), in a 15.2 km^2^ area dominated by a 128.9 ha glyphosate-resistant Roundup Ready® alfalfa seed production field and several non-Roundup Ready alfalfa seed production fields (totaling 120.2 ha). Each year, honey bee self-marking devices were placed on 112 selected honey bee colonies originating from nine different apiary locations. The foraging bees exiting each apiary location were uniquely marked so that the apiary of origin and the distance traveled by the marked (field-collected) bees into each of the alfalfa fields could be pinpointed. Honey bee self-marking devices were installed on 14.4 and 11.2% of the total hives located within the research area in 2006 and 2007, respectively. The frequency of field-collected bees possessing a distinct mark was similar, averaging 14.0% in 2006 and 12.6% in 2007. A grand total of 12,266 bees were collected from the various alfalfa fields on seven sampling dates over the course of the study. The distances traveled by marked bees ranged from a minimum of 45 m to a maximum of 5983 m. On average, marked bees were recovered ∼ 800 m from their apiary of origin and the recovery rate of marked bees decreased exponentially as the distance from the apiary of origin increased. Ultimately, these data will be used to identify the extent of pollen-mediated gene flow from Roundup Ready to conventional alfalfa.

## Introduction

Alfalfa (*Medicago sativa* L.) is one of the most important crops grown in the United States that requires bee pollination for seed production (Van Deynze et al. 2008; Orloff et al. 2009; Cane 2002). Specifically, alfalfa is a cross-pollinated, perennial crop that requires bees to “trip” flowers to release pollen for seed production (McGregor 1976). Typically, alfalfa seed producers depend on honey bees *Apis mellifera* L. (Hymenoptera: Apidae), alfalfa leafcutting bees *Megachile rotundata* (F.) (Hymenoptera: Megachilidae), and/or alkali bees *Nomia melanderi* Cockerell (Hymendoptera: Halictidae) to pollinate their fields. As such, alfalfa seed producers rent bees from beekeepers and strategically place the hives next to their blooming alfalfa fields to maximize pollination and subsequent seed yield.

Recent advances in biotechnology offer opportunities for improvement of alfalfa production. Roundup Ready (Monsanto, www.monsanto.com) alfalfa is the first transgenic crop grown as a perennial to contain the gene for tolerance to glyphosate. Its introduction in 2005 was controversial because the mitigation of gene flow (the unintentional movement of the Roundup Ready gene to non-Roundup Ready alfalfa plants) is more complex with perennial crops than with annual crops (Chandler and Dunwell 2008). The environmental consequences of unintended gene flow of genetically engineered traits include transfer of the trait to related plants, increasing their potential to become weeds (e.g., emergence of volunteer plants), as well as transfer of the trait to conventional and/or organic crops, limiting their acceptance in sensitive markets (Van Deynze et al. 2008). The major economic consequence is the potential for lost sales due to the admixture of the genetically engineered trait in non-genetically engineered seed (Mallory-Smith and Zapiola 2008). To this end, knowledge of honey bee foraging behavior and the extent of pollen-mediated gene flow between commercial alfalfa seed production fields are needed to minimize adventitious presence of the modified gene in conventional alfalfa fields. The goal of this study is to quantify honey bee dispersal patterns throughout a commercial alfalfa seed production area that contains both Roundup Ready and conventional alfalfa. To accomplish this goal, honey bee self-marking devices loaded with various distinct powdered markers were placed at the entrances of 112 honey bee hives, located in nine apiaries within a 15.2 km^2^ study area dominated by alfalfa seed production fields (Hagler et al. 2011). The bees exiting each apiary were uniquely marked so that the apiary of origin and the distance traveled by the marked (field-collected) bees could be pinpointed. Ultimately, these data will be correlated with seed harvest data to identify the extent of pollen-mediated gene flow from Roundup Ready to conventional alfalfa.

## Materials and Methods

### Study site

**Figure 1.**
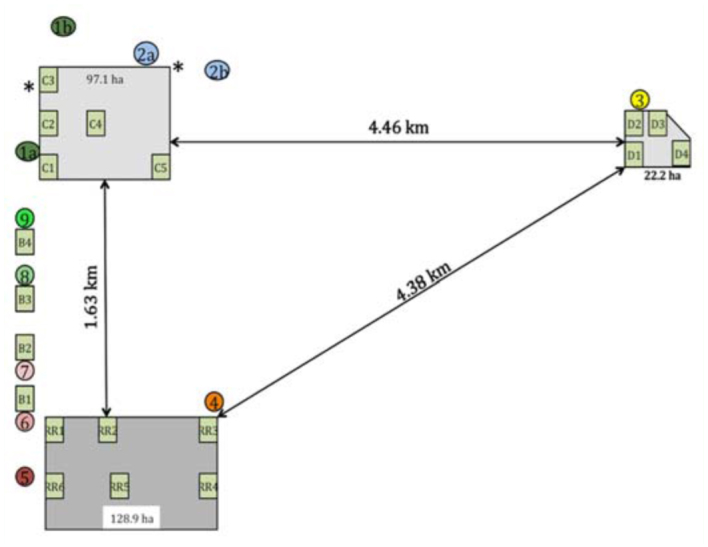
Diagrammatic representation of the study site located in Fresno County, CA. The large dark grey rectangle represents a 128.9 ha Roundup Ready alfalfa field, the large light grey rectangle represents a 97.1 ha conventional alfalfa field, and the large light grey polygon represents a 22.2 ha conventional alfalfa field. The small green rectangles designated as B1, B2, B3, and B4 represent four small conventional fields. All the small green rectangles represent the 19 areas from which honey bees were collected; see Figure 2 for a depiction of the sampling scheme. The variously colored circles represent the locations of nine honey bee apiaries placed adjacent to the alfalfa seed fields by beekeepers for pollination. Note that apiary 1 and 2 consisted of two separate groups (bee drops) of hives that contained the same mark (green and blue colored powder, respectively). The GPS coordinates for the point of origin of the marked bees for apiaries 3 through 9 were at the center of each apiary. The GPS coordinates for the point of origin of the marked bees originating from apiaries 1 and 2 were set at the half way point between the honey bee drop zones. These points are indicated by an asterisk. The number of hives, the number of marked hives, and the specific mark(s) placed in each apiary is given in Table 1. High quality figures are available online.

A schematic diagram of the 15.2 km^2^ study site is shown in Figure 1. The study was conducted in an alfalfa seed production area located in Fresno County, CA, USA, during alfalfa bloom in 2006 and 2007. The area contained seven alfalfa fields from which foraging honey bees were collected. The seven fields included one 128.9 ha transgenic herbicide-tolerant seed production field (hereafter referred to as the RR field), four small 0.73 ha conventional alfalfa seed production fields (hereafter referred to as Conventional B fields 1, 2, 3, and 4), one 97.1 ha conventional alfalfa seed production field (hereafter referred to as the Conventional C field), and one 22.2 ha conventional alfalfa seed production field (hereafter referred to as the Conventional D field). It should be noted that the RR field and the Conventional C and D fields were established commercial fields. The four Conventional B fields were strategically planted in an equidistant linear fashion along the west edge between the RR field and the large Conventional C field to serve as a “bridge” between the two types of commercial alfalfa seed. Each alfalfa field, depending on its size, contained one to six 0.73 ha honey bee collection sites (Figure 1). The 128.9 ha RR field was the only source of genetically engineered alfalfa within the study area for at least 10 km in any direction. The Conventional B, C, and D fields were the only conventional alfalfa fields in the vicinity. A plant habitat survey of the area revealed that there were very few honey bee attractive crops such as cotton, onion, garlic, tomato, wheat, oats and beets in the surrounding landscape.

A total of 776 and 1000 commercial honey bee colonies (2- to 4-story Langstroth hives) were placed in nine apiary locations within the study area in 2006 and 2007, respectively, when the fields were in early bloom (33–50% bloom). The relative location of each apiary is shown in Figure 1. The total number of hives, the number of marked hives, and the type of mark placed at each apiary are given in Table 1. The number of honey bee colonies placed near each of the fields was based roughly on the industry standard of 4.9–7.4 honey bee colonies per hectare for optimal alfalfa seed production (Mueller 2007). Therefore, the RR field and the Conventional C and D fields had hundreds of honey bee hives placed at each apiary location, whereas each of the Conventional B fields had four honey bee hives.

### Honey bee marking procedure

A more thorough description of the honey bee marking procedure is described by Hagler et al. (2011). In brief, a small self-marking device was attached at the entrance of 112 honey bee hives on 28 June 2006 and 18 June 2007, respectively. The remaining portion of the hive entrance was blocked with either nylon or wire screen to ensure that the only passageway into or out of the hive was through the 73 mm opening of the marking device. The bees were given 20–44 hours to adjust to the alteration of the hive entrance. Then, on each honey bee sample collection date, a 50 mL tube containing one of nine distinct powdered marks was inserted into each of the marking devices between 06:00 and 07:00 (prior to the initiation of honey bee flight). The device administered a mark on bees as they exited the hive. This method facilitated the synchronous and distinct marking of thousands of foraging honey bees exiting each apiary. The marks consisted of either one of five fluorescent-colored DayGlo™ powders (green, blue, yellow, orange or magenta (DayGlo, www.dayglo.com) or a combination of green or magenta colored powder mixed (1:1) with either dry bovine milk powdered casein (Dairy America Inc., www.dairyamerica.com) or chicken egg white powder egg albumin (Barry Farm Foods, www.barryfarm.com) protein. The distinct mark(s) assigned to each apiary is given in Table 1. It was not feasible to mark every hive within the large study site. Therefore, 8 to 15.4% of the bee hives in the large apiaries and all the hives in the small apiaries were fitted with a marking device.

**Figure 2.**
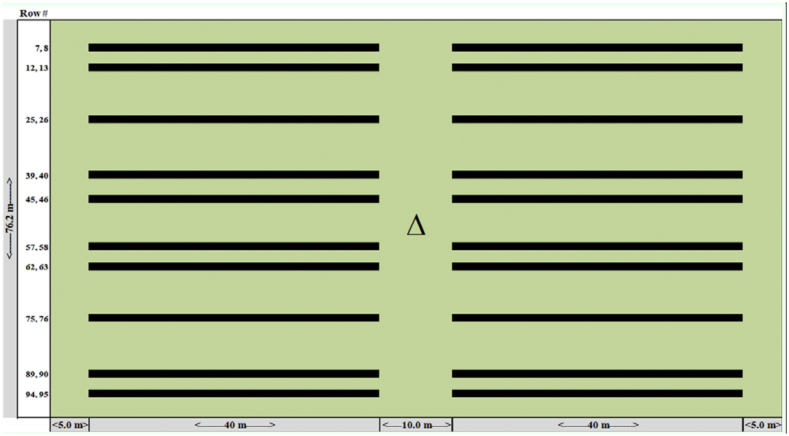
Diagrammatic representation of the location (rows) where 40 m sweep samples (black rectangles) were taken at each of the 19 sample sites (n = 20 per plot). The triangle represents the center of each sampling site where the GPS coordinates were used to determine the honey bee foraging range. High quality figures are available online.

### Honey bee sampling procedure

Foraging honey bees were collected within the nineteen 0.73 ha pre-designated sampling sites in the various alfalfa fields, using 38 cm diameter sweep nets. There were six sampling sites in the RR field, five in the Conventional C field, and four in the Conventional D field. Each of the four small Conventional B fields only contained one sample site, which comprised the entire field (Figure 1). Samples were collected on 29 June, 30 June, and 18 July 2006, and 20 June, 21 June, 11 July, and 12 July 2007 when each field was at full bloom and the honey bees were actively foraging. Generally, honey bees were collected at each site on each of the above dates. However, on 29 June 2006 there were no samples collected in the RR field due to a time constraint, and in a few instances some sampling sites were not accessible because the fields were being irrigated. A diagram of the sampling scheme used at each sample site is given in Figure 2. Each sampling site consisted of a 76.2 m wide (100 – 76.2 cm rows) by 96.6 m long (0.73 ha) alfalfa plot (note: the exact size of the Conventional B fields). Twenty 40 m sweep samples, bridging two rows, were collected from each sampling site on each sampling date. A systematic sampling procedure was used throughout the study to collect pollinator samples from areas adjacent to where seed samples would be collected at harvest (Teuber et al., unpublished). Two samples were taken along rows: 7 and 8, 12 and 13, 25 and 26, 39 and 40, 45, and 46, 57 and 58, 62 and 63, 75 and 76, 89 and 90, and 94 and 95 (Figure 2). Sweep samples were collected as the sampler walked between pre-designated rows; when the sampler was ∼ 5.0 m into the field, he/she took 20 sweeps bridging the two rows while walking at a continuous pace of ∼ 1.0 m/sec. After the sampler had walked 40 m, the contents of the sweep net were placed into a plastic bag, pre-labeled with the date and location of the sample. The bag was sealed and tightly rolled to minimize the movement of the bees within the bag. The sampler then proceeded to the next sample location by walking forward ∼ 10 m from the termination point of the first sweep sample and sweeping another 20 times within a distance of 40 m as described above. The sampler then walked straight out of the remaining portion of the sample area (∼ 5.0 m), walked across to the next pre-designated sample row pair, and continued the process described above in the opposite direction, returning to the edge of the field. In all, there were five pairs of samplers, so that each 0.73 ha sampling area could be sampled in under 15 min. When the samplers completed their round, the samples were immediately placed in an ice chest containing dry ice to immobilize the bees. Honey bees were collected during peak flight activity between 08:00 and 14:00 on each sample date. The first site sampled each day was chosen at random and then the subsequent sampling sites were sampled in clockwise order to expedite the laborious collection process. All samples were placed into a -20 °C freezer at the laboratory and held until analyzed for the presence of marks.

### Detection of marks on field-collected honey bees

Field-collected bees were removed from the freezer and examined individually under ultraviolet light and a 10× dissecting microscope for the presence of colored fluorescent powder. Each bee was scored as either positive or negative for the presence of fluorescent powder. If a colored powder was detected on a bee, the color was recorded and a numerical score was assigned based on the amount of powder observed. A marked bee scored “1” if just a few grains of powder were observed (e.g., it took some time to locate the powder on the bee under UV light and the dissecting microscope), “2” if a moderate amount of powder was observed (e.g., immediately distinguishable under UV light and the microscope), and “3” if the bee was heavily powdered (e.g., immediately distinguishable under UV light only). There was a slight chance that an unmarked bee could be contaminated with a mark during the sampling process, by direct contact of an unmarked bee with a marked bee in the sample bags. Therefore, to minimize the possibility of including falsely marked bees in our assessment, we only included bees that scored 2 or 3 in the final data analysis. In order to achieve nine distinct marks within the study area, our marking scheme included double marks on bees exiting the four Conventional B field apiaries (see Table 1). Therefore, every bee that contained a green or magenta mark was also analyzed for the presence of egg albumin and milk casein protein by protein-specific enzyme-linked immunosorbent assays (ELISA) to clarify its apiary of origin. In other words, if a bee contained magenta or green powder plus one of the protein marks, it originated from one of the four Conventional B fields; if it contained only green or magenta powder, it originated from apiary 1 or 5, respectively. Magenta and green marked bees were placed individually into 1.5 mL microcentrifuge tubes containing 1000-µL of tris-buffered saline (TBS, pH 7.4), soaked for ≥ one hour with constant agitation (120 rpm), and analyzed for the presence of each protein mark by the ELISAs described by Hagler et al. (2011).

### Honey bee negative controls

Honey bees serving as negative controls (n = 8 per ELISA plate) were collected from colonies maintained at the USDA-ARS, Carl Hayden Honey Bee Research Laboratory, Tucson, AZ, USA. Negative control bees were visually examined as described above for the presence of fluorescent powders and then assayed for the presence of egg albumin and milk casein protein marks by the protein-specific ELISAs. Mean (±SD) ELISA optical density values were calculated for the negative control bees. Field-collected bees were conservatively scored positive for each protein mark if the ELISA optical density value was six standard deviations above that of the negative control mean.

### Data analysis

The total number of honey bees collected and the number of honey bees containing a specific mark(s) in each of the 19 sampling areas was tallied. Based on its distinct mark, the distance that each bee traveled from its apiary of origin was precisely calculated with ArcMap 9.2 (ESRI, www.esri.com) using the GPS coordinates of the apiary possessing the specific mark (its point of origin) and the coordinates of the middle of the sampling area from which it was collected. The average (±SD), minimum, and maximum distances that the marked bees traveled from each apiary were determined. Data were sorted by sample date and pooled within field sites to simplify the data presentation.

The distances that the field-collected bees traveled from the apiaries placed adjacent to the RR field (e.g., those with an orange or magenta only mark) were fitted with a negative exponential equation (SigmaPlot 11.0, www.sigmaplot.com) to describe their foraging range distribution. The number of marked bees collected on each sample date was pooled for both years to provide a more robust analysis of the dispersal distance. No orange or magenta marked bees were collected in the Conventional D field, and were therefore omitted from the analysis.

## Results

**Figure 3.**
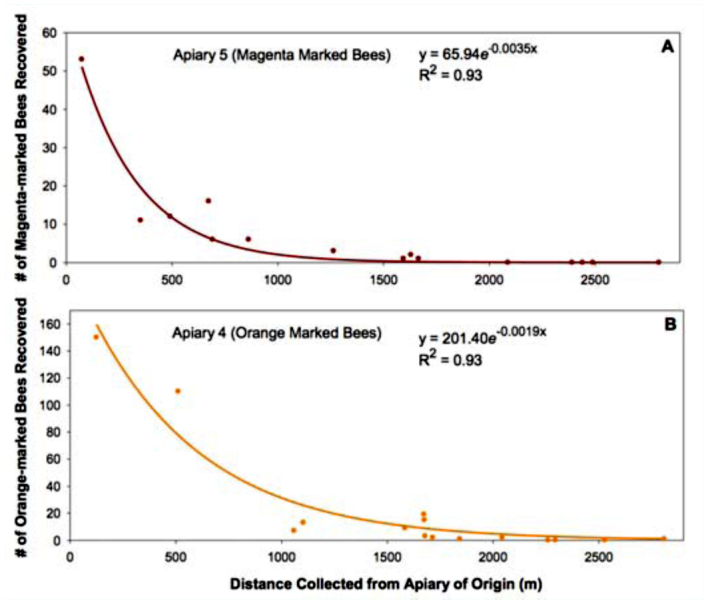
Relationship between the distance that a marked bee was collected from her apiary of origin (x axis) and the total number of marked bees collected in blooming alfalfa fields. (A) Bees originating from the apiary singly marked with magenta colored powder (apiary S) and (B) bees originating from the apiary singly marked with orange colored powder (apiary 4). The location of the apiaries of origin, apiaries 5 and 4, respectively are given in Figure 1. High quality figures are available online.

Honey bee self-marking devices were installed on 14.4 and 11.2% of the hives located within the 15.2 km^2^ alfalfa seed production study area in 2006 and 2007, respectively (Table 1). The frequency of fieldcollected bees possessing a distinct mark was similar, averaging 14.0% in 2006 and 12.6% in 2007 (Table 2). These data indicate that the marking devices placed at the entrances of selected hives effectively delivered a powdered mark to bees exiting those hives. The hives in each apiary contained a distinct mark, which enabled identification of the apiary of origin and distance traveled by each marked field-collected honey bee. A grand total of 12,266 bees (4,391 in 2006 and 7,875 in 2007) were collected on seven sampling dates over the course of the two year study (Table 2). The distribution of bees over the study area was fairly uniform, with an overall average of 4.32 ± 4.6 and 5.79 ± 7.3 bees collected in each field during 2006 and 2007, respectively. Travel distance by marked bees ranged from a minimum of 45 m (the closest collection point from an apiary) to a maximum of 5983 m. On average, marked bees were recovered 738.1 ± 732 m and 864.9 ± 919 m from their apiary of origin in 2006 and 2007, respectively (Table 2). As expected, the vast majority of marked bees collected in the various fields were from those apiaries closest to the sampling site within that field. Since this study was part of a much larger study focusing on pollen-mediated gene flow from the genetically engineered alfalfa seed field, special attention was paid to honey bee movement from apiaries located near the RR field to the conventional fields in the study area. The dispersal distance of the fieldcollected bees originating from the apiaries adjacent to the RR field (bees possessing an orange or magenta mark) was best described by a negative exponential decay equation (Figure 3). Specifically, the recovery rate of orange or magenta-marked bees decreased exponentially as the distance from the apiary of origin increased. The recovery rate of the marked bees originating from apiaries placed near the conventional fields displayed a similar foraging range distribution (data not shown).

## Discussion

It is well known that honey bees can travel more than 10 km in search of desirable floral rewards. A small number of “scout” bees tend to fly the longer distances (Vansell and Todd 1946; Levin et al. 1960; Bradner et al. 1965; Ramsay et al. 2003; Ramsay 2005; Chandler and Dunwell 2008; Gary 1992; Williams 2001; Visscher and Seeley 1982). These extreme flight distances suggest the existence of a maximum potential distance that pollen-mediated gene flow can occur in a crop that is dependent on bees for pollination. However, it is also well known that bees tend to forage within 2.0 km of their hive if there are attractive floral resources in the vicinity (Pedersen et al. 1972; Osborne et al. 2001). The dominant honey bee attractive flora in bloom during this study was alfalfa. Other crops in the vicinity included cotton, onion, garlic, sugar beets, tomato, wheat, and oats. While honey bees are known to visit some of these crops, they are generally not regarded as highly attractive pollen or nectar resources for bees (McGregor 1976). As such, this study design allowed us to evaluate the “worst case scenario” in terms of assessing the potential for unintentional gene flow in alfalfa (Teuber et al., unpublished). Our study revealed that for alfalfa, the number of honey bees foraging away from the hive decreases exponentially with distance (Figure 3). On average, honey bees travelled 738 and 865 m from their apiary in 2006 and 2007, respectively. However, a maximum foraging distance of 5.98 km was recorded. The abundance of blooming alfalfa, which is typical of seed production fields, kept the honey bee foraging distances observed during this study to a minimum. Furthermore, the lack of highly attractive crops in the vicinity likely restrained bees from foraging far from hives. The presence of other attractive crops like sunflowers might have drawn bees further from the hives and reduced foraging (thus gene flow) on alfalfa.

Given the information above, identifying environmentally acceptable isolation distances between transgenic and conventional alfalfa seed production fields is difficult, because the maximum dispersal range of an individual bee does not necessarily correlate to successful gene flow (e.g., pollen transfer resulting in viable seed set). Along with foraging distance, the number of individual flowers visited and the spatial scale of the visits by a bee on any given foraging trip will dictate the degree of gene flow between RR and conventional alfalfa (Pedersen 1953; Teuber et al. 1983; Cane 2002). Also, an alfalfa flower must be tripped in order to be successfully pollinated. Among the primary pollinators of alfalfa, honey bees are among the least efficient at tripping alfalfa. Female alkali bees and alfalfa leafcutting bees have a tripping efficiency of about 80%, while honey bees only trip about 22% of the flowers they visit (Cane 2002).

This study was designed to identify the honey bee foraging patterns in a large alfalfa seed production area in California, containing both RR and conventional alfalfa seed fields. Ultimately, the pollen-mediated gene flow from the RR source field to the conventional alfalfa fields will be quantified by using the RR trait as a genetic marker (Teuber et al., unpublished). Specifically, alfalfa seed harvested from the sampling sites in the conventional alfalfa fields will be grown and sprayed with Roundup® herbicide when they reach the appropriate stage of development. The number of seedlings surviving the herbicide treatment will provide a direct measurement of RR gene flow. Further confirmation that the RR trait is present in the surviving plants will be determined by a RR-specific immunoassay (Messeguer et al. 2004; St. Amand 2000; Umbeck et al. 1991; Van Deynze et al. 2005;). Given all of the parameters associated with effective pollen transfer by bees including distance, frequency of floral visits, “tripping” efficiency, etc., it is unlikely that significant gene flow from a large RR source alfalfa field to a conventional field will extend beyond the 1.5 km reported in a smaller study by Teuber et al. (2005).

In summary, understanding gene flow mediated by honey bee pollen dispersal is crucial for developing strategies to minimize adventitious presence of a genetic trait. The data described here on the foraging range of honey bees, coupled with analysis of the seed harvested from the study site, will help establish isolation requirements to ensure genetic purity of alfalfa seed. Many key questions that were beyond the scope of this study need to be addressed in the near future. For example, do bees move from source to source when they forage, causing a bridging effect in gene flow, or do they go from a source of pollen directly back to the hive? Does wind or other topographical cues affect honey bee behavior, and thereby influence gene flow? What are the dynamics of gene flow, as it relates to bee foraging both into and away from fields planted with transgenic cultivars? Does the relative size of the field affect foraging? Understanding how genes move via honey bee-carried pollen is crucial for developing strategies to minimize adventitious gene presence and assess novel trait environmental impact.

**Table 1.**
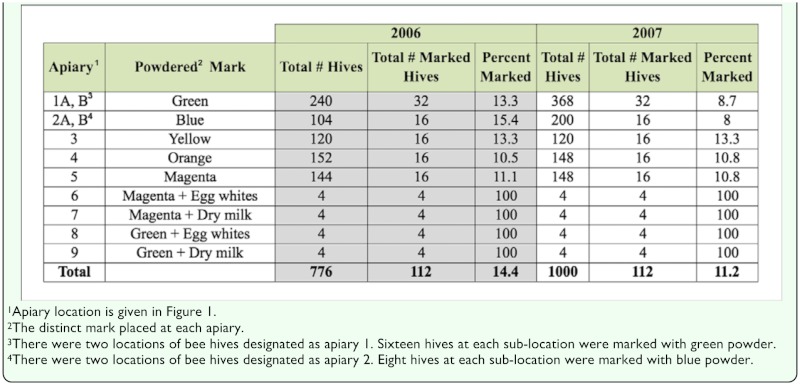
A summary of the honey bee colony demographics at each of nine apiaries during the 2006 and 2007 studies.

**Table 2.**
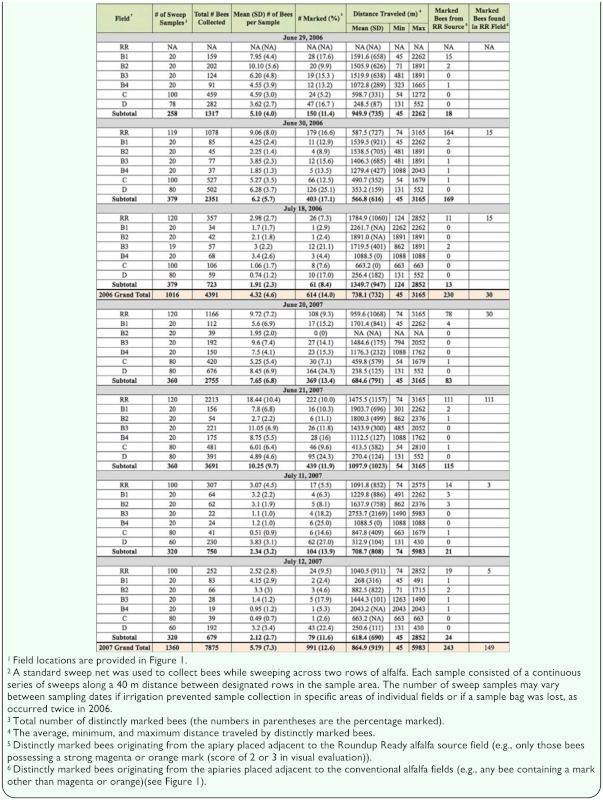
A summary of bees collected from sweep samples by date and field. All sampling areas were combined within each field. Only bees with a strong mark from their apiary of origin were used for distance calculations. Distances were measured from the center of the apiary to the center of the sweep sampling area.
